# Six new deep-water sternaspid species (Annelida, Sternaspidae) from the Pacific Ocean

**DOI:** 10.3897/zookeys.348.5449

**Published:** 2013-11-08

**Authors:** Sergio I. Salazar-Vallejo, Galina Buzhinskaja

**Affiliations:** 1 El Colegio de la Frontera Sur, CONACYT, Chetumal, México; 2 Zoological Institute, Russian Academy of Sciences, Saint-Petersburg, Russia

**Keywords:** Polychaete, taxonomy, systematics, introvert color, shield fan, shield development

## Abstract

Most sternaspid species have been described from shallow water, and *Caulleryaspis* Sendall & Salazar-Vallejo, 2013 includes one deep water species: *C. gudmundssoni* Sendall & Salazar-Vallejo, 2013 from Iceland. In *Sternaspis* Otto, 1821, the most speciose genus, most species were described from shallow water and only three thrive in deep water: *S. maior* Chamberlin, 1919 from the Gulf of California, *S. princeps* Selenka, 1885 from New Zealand, and *S. riestchi* Caullery, 1944 from Indonesia. The study of some deep sea sternaspids from the Pacific Ocean in the collections of six research institutions resulted in the discovery of six undescribed species, and for three of them there were abundant materials showing ventro-caudal shield development. *Caulleryaspis fauchaldi* **sp. n.** is described based on specimens from Oregon and California; it differs from the known species because it has a shield with rounded anterior margins and its peg chaetae form thin, small spines. *Caulleryaspis nuda* **sp. n.** was collected off Oregon; it is unique because its shield lacks a layer of sediment particles firmly attached, but has instead a thin layer of small particles loosely attached. Four other species are newly described in *Sternaspis*: *S. annenkovae* **sp. n.** was collected east off the northern Kurile Islands in about 4,000 m depth; it differs from other species by having a bicolored body, with the introvert darker than the abdomen, and its ventro-caudal shield plates are divergent resulting in a divided fan. The second species, *S. maureri* **sp. n.** was found off Peru in 1296–6489 m water depths and in the Southwestern Pacific in 795–3830 m; it resembles *S. williamsae* **sp. n.** but differs because its shield has better-developed ribs, the fan has a shallow or indistinct median notch and has lateral notches well-developed. The third species, *S. uschakovi* **sp. n.**, was found in the Okhotsk Sea in 592–1366 m, off California in 1585 m, Gulf of California in 1200–1274 m, and Western Mexico in 2548 m; it resembles *S. africana* Augener, 1918 and *S. andamanensis* Sendall & Salazar-Vallejo, 2013 in having shields with a denticulate posterior margin; the latter two species live in shallow water and they differ from each other and from the new species by a combination of shield and papillae features. The fourth species, *S. williamsae* **sp. n.**, was found off Oregon in 1000–2400 m, and off California in 878–1246 m; it resembles *S. annenkovae* because both species have shields with fans narrower than the anterior margin width, but differ in the relative development of shield features and in the relative size of prostomium and mouth; as stated above it also resembles *S. maureri* **sp. n.** but its shield has poorly-developed ribs, its median notch is distinct, and the lateral notches are poorly developed or indistinct. Keys to identify all species of *Caulleryaspis* and *Sternaspis* are also included.

## Introduction

In the polychaete family Sternaspidae Carus, 1863, most species have been described from shallow water, of less than 200 m depth (Sendall and Salazar-Vallejo 2013); however, one of the two known species of *Caulleryaspis* Sendall & Salazar-Vallejo, 2013 and only three species in *Sternaspis* Otto, 1821 were described from deeper water, of around 1000 m depth: *Sternaspis maior* Chamberlin, 1919, *Sternaspis princeps* Selenka, 1885, and *Sternaspis rietschi* Caullery, 1944. The former was described from the Gulf of California, Eastern Pacific Ocean, whereas the two other species were described from specimens collected in the Southwestern Pacific. Deep water sternaspids are delicate, fragile and difficult to study because they are often rare, but some abundant specimens have allowed us to study the development of the ventro-caudal shield.

The study of the Pacific Ocean sternaspid material lodged in six major research institutions resulted in the recognition of six species which are newly described: *Caulleryaspis fauchaldi* sp. n. from off Oregon and California, *Caulleryaspis nuda* sp. n. from off Oregon, *Sternaspis annenkovae* sp. n. from off the Northern Kurile Islands, *Sternaspis maureri* sp. n. from off Peru and from the Southwestern Pacific, *Sternaspis uschakovi* sp. n. from the Okhotsk Sea, California, Gulf of California and Western Mexico, and *Sternaspis williamsae* sp. n. from off Oregon and California.

## Methods

Specimens were cleaned with a small brush; measurements were made with a millimeter ruler and the ventro-caudal shield was measured with a micro ruler with 0.1 mm marks. Ventro-caudal shield fascicles were counted by viewing the chaetal bases end on because most were broken. Collapsed specimens were re-swollen by injecting the same preserving fluid with a syringe. Illustrations were prepared by assembling series of photographs by using HeliconFocus. Character choice and terminology follows a recent revision (Sendall and Salazar-Vallejo 2013; species differentiation is especially based upon shield differences. Station data of the R.V. Anton Bruun cruise 11 are after Menzies and Chin (1966), and Maurer and Williams (1988). Specimens belong to the following institutions:

CAS California Academy of Sciences, San Francisco

LACM Natural History Museum of Los Angeles County, Allan Hancock Polychaete Collection, Los Angeles

SIORAS Shirshov Institute of Oceanology, Russian Academy of Sciences, Moscow

UNAM Colección de Referencia de Invertebrados Bentónicos, Unidad Académica Mazatlán, UNAM, Mazatlán

ZIRAS Zoological Institute, Russian Academy of Sciences, Saint-Petersburg

ZMUC Zoological Museum, University of Copenhagen

## Systematics

### Sternaspidae Carus, 1863

#### Caulleryaspis

Sendall & Salazar-Vallejo, 2013

http://species-id.net/wiki/Caulleryaspis

##### Type species.

*Caulleryaspis gudmundssoni* Sendall & Salazar-Vallejo, by original designation.

##### Diagnosis (emended).

Sternaspids with introvert hooks falcate, tapered. Pre-shield region with 7 segments. Ventro-caudal shield flexible, usually with abundant sediment particles firmly adhered, rarely sediment particles loosely adhered; without well-defined radial ribs and concentric lines. Branchial filaments arranged in discrete branchial plates.

#### Caulleryaspis fauchaldi sp. n.

http://zoobank.org/C35D7377-3C94-49A6-B124-98873D0BB7A5

http://species-id.net/wiki/Caulleryaspis_fauchaldi

Figures 1 , 2

Sternaspis fossor : Hartman 1963: 59; Fauchald and Hancock 1981: 35 (*partim*, *non*Stimpson 1853).

##### Type material.

**Northeastern Pacific, Oregon**. Holotype (LACM 5360), and paratype (LACM 5361), Cascadia Abyssal Plain, west of Yaquina Bay, R.V. Acona, Sta. AD 33, NAD 21 (44°30.0'N, 125°34.0'W – 44°39.0'N, 125°33.2'W), 2800 m, clayey silt, 25 Jan. 1963 (paratype breaking into two parts, 8.5 mm long, 4 mm wide, introvert not exposed; left shield plate 2.3 mm long, 1.5 mm wide).

##### Additional material.

**Northeastern Pacific. Oregon.** Four specimens (CAS 128953f), variably damaged, Sta. BMT 556 (48°7.7'N, 127°4.8'W), 2519 m, 10 Sep. 1971, A. Carey, coll. (9–10 mm long, 4.0–4.5 mm wide; left shield plate 2.2–2.3 mm long, 2.4–2.5 mm wide). One specimen (CAS 129027f), Sta. BMT 557 (48°9.0'N, 127°4.2'W), 2519 m, 10 Sep. 1971, A. Carey, coll. (9.5 mm long, 4 mm wide; left shield plate 2.5 mm long, 2.9 mm wide). **Southern California canyons.** One specimen (LACM 5362), 8.1 km off Pyramid Head, Lighthouse, San Clemente Island, R.V. Velero IV, Sta. 6839 (32°46'30"N, 118°15'43"W), 1387 m, coarse sand, 30 Jun. 1960 (3.2 mm long, 1.8 mm wide, abdomen 2.5 mm long; left shield plate 0.8 mm long, 0.8 mm wide). **BLM (Bureau of Land Management), Baseline Study, Southern California Bight**. One specimen (LACM 5333), dried-out, BLM 81304, R.V. Thomas G. Thompson, Sta. 813 (33°0.903'N, 119°2.188'W), 1753 m, olive brown silt, biological box core, rep. 04 BFI/ B4-1, 23 Aug. 1977, K. Fauchald, G. Jones, coll. One specimen (LACM 5335), dried-out, BLM 81308, R.V. Thomas G. Thompson, Sta. 813 (33°0.933'N, 119°1.919'W), 1753 m, olive brown silty mud, biological box core, rep. 08 BFI/ B4-1, 23 Aug. 1977, K. Fauchald, G. Jones, coll. One specimen (LACM 5337), dried-out, BLM 81352,, R.V. Thomas G. Thompson, Sta. 813 (33°1.05'N, 119°1.96'W), 1723 m, olive brown mud, biological box core, rep. 52 BFI/ B4-1, 31 Aug. 1977, K. Fauchald, G. Jones, coll.

##### Description.

**Figure 1.**
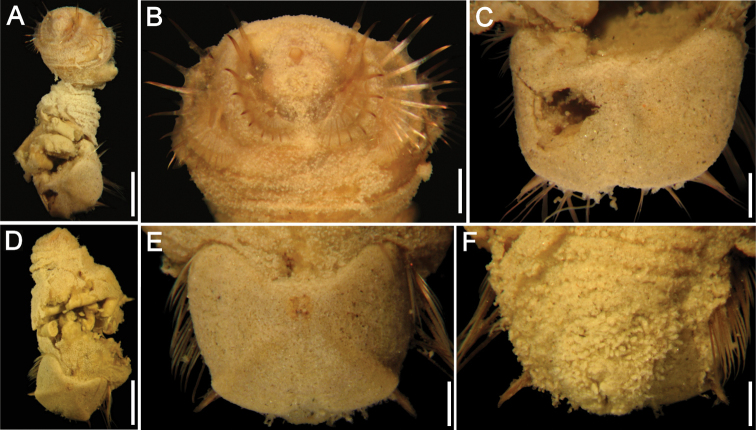
*Caulleryaspis fauchaldi* sp. n. **A** Holotype (LACM 5360), ventral view **B** Anterior end, frontal view **C** Ventro-caudal shield, frontal view **D** Paratype (LACM 5361), ventral view **E** Ventro-caudal shield, frontal view **F** Posterior end, dorsal view. Bars: **A** 1.8 mm **B, C, E, F** 0.6 mm **D** 2 mm.

Holotype (LACM 5360) and paratype breaking into two pieces (Fig. 1A, D). Body grayish, with introvert exposed, slightly darker, broken dorsally; abdomen breaking ventrally, ventro-caudal shield grayish. Integument papillae abundant, shorter on introvert, larger on abdomen, incorporating sediment particles forming a thick coat over most of body (including introvert in paratype), arranged in single transverse series in posterior chaetigers. Body 9 mm long, 3 mm wide, about 28 segments.

Prostomium minute globose, ovoid, brownish (Fig. 1B). Peristomium small, oval, bearing abundant papillae resembling those present over introvert, extended as a wide band over prostomium. Mouth oval, small, slightly projected, covered by papillae.

First three chaetigers with about 16 falcate, tapered introvert hooks per bundle, each with subdistal dark areas (tips broken, darker areas look distal; subdistal in complete hooks). Genital papillae not seen (paratype with ventrolateral pores between segments 7 and 8). Pre-shield region with 7 segments (difficult to count because of specimens fragility); capillary chaetae along first pre-shield segment (paratype with capillaries in two segments).

Ventro-caudal shield completely covered by a thick coating of adhered particles (Fig. 1C), perforated, better preserved in paratype (Fig. 1E); suture not visible. Anterior margins clearly rounded; anterior depression deep; anterior keels not exposed. Ribs or concentric lines not visible. Lateral margins rounded, expanded medially, reduced posteriorly. Fan truncate, barely reaching posterior corners. Other features not visible.

Marginal chaetal fascicles include 9 lateral and only 4–5 short, small posterior ones (others probably broken), each with 3–4 chaetae per bundle. Peg chaetae robust, forming thin, short spines, close to posterior margins. Additional chaetal fascicles not visible.

Branchiae lost. Interbranchial filaments lost. Branchial plates slightly divergent, anteriorly expanded, rounded (Fig. 1F).

**Figure 2.**
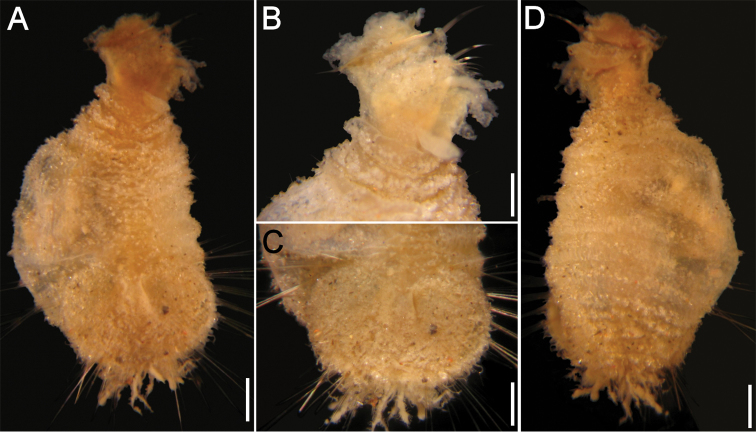
*Caulleryaspis fauchaldi* sp. n., juvenile specimen (LACM 5362) **A** Ventral view **B** Anterior end, ventral view **C** Ventro-caudal shield, frontal view **D** Dorsal view. Bars: **A, C, D** 0.38 mm **B** 0.26 mm.

**Juveniles**. Juvenile (Fig. 2A) with papillae less abundant and larger than those present in type specimens, homogeneously distributed throughout integument, eroded in introvert and arranged in transverse series as remains of erosion along dorsal surface (Fig. 2D). Body about one-third as large as type specimens, with introvert damaged by compression (Fig. 2B). Ventro-caudal shield with sediment particles and abundant papillae (Fig. 2C); anterior margins poorly defined, lateral margins rounded, medially expanded. About 9 lateral chaetal bundles and 5–6 posterior ones with longer, thinner and fewer chaetae than in larger specimens. Peg chaetae not visible.

##### Etymology.

This species is named after Dr. Kristian Fauchald, long-time teacher and friend, in recognition of his many contributions to polychaete systematics and especially because of his contribution to the study of deep-sea fauna including the off Oregon species. The epithet is a noun in the genitive case.

##### Type locality.

W off Yaquina Bay, 2800 m depth.

##### Remarks.

*Caulleryaspis fauchaldi* sp. n. is very similar to *Caulleryaspis gudmundssoni* Sendall & Salazar-Vallejo, 2013 because both have shields with a deep anterior depression and robust peg chaetae. These two species differ, however, in two main features. In *Caulleryaspis fauchaldi* the anterior shield margins are rounded, the introvert has longer papillae, and the peg chaetae form thin short spines, whereas in *Caulleryaspis gudmundssoni* the anterior shield margins are more angular, the introvert has shorter papillae, and the peg chaetae form thick, long spines.

##### Distribution.

From Oregon to Southern California, in 1387–2800 m depth.

#### Caulleryaspis nuda sp. n.

http://zoobank.org/B3C65AD6-0C77-421C-8CFB-8E9C724866D0

http://species-id.net/wiki/Caulleryaspis_nuda

Figure 3

##### Type material.

**Northeastern Pacific, Oregon**. Holotype (CAS 129027h) and nine paratypes (CAS 129027p), variably damaged, Sta. BMT 557 (48°9.0'N, 127°4.2'W), 2519 m, 10 Sep. 1971, A. Carey, coll. (complete paratypes 9–11 mm long, 3–5 mm wide; left shield plate 1.8–2.1 mm long, 1.9–2.4 mm wide; oocytes 200 µm in smaller paratype).

##### Additional material.

**Northeastern Pacific, Oregon.** Thirty-three specimens (CAS 128953), Sta. BMT 556 (48°7.7'N, 127°4.8'W), 2519 m, 10 Sep. 1971, A. Carey, coll. (complete 8.0–11.5 mm long, 3.5–5.5 mm wide; left shield plate 1.5–2.5 mm long, 1.5–2.4 mm wide; oocytes 200 µm).

##### Description.

**Figure 3.**
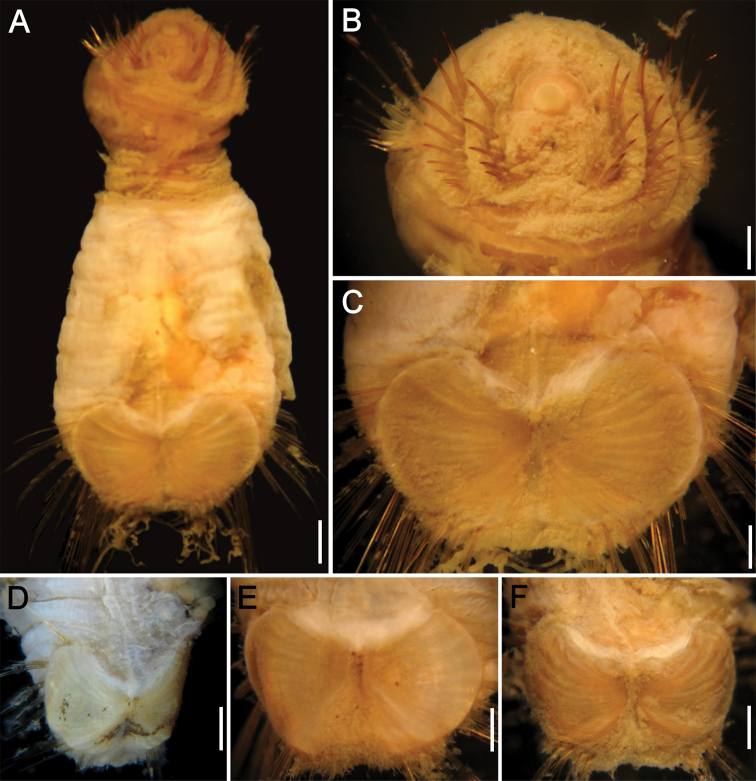
*Caulleryaspis nuda* sp. n., **A** Holotype (CAS 129027h), ventral view **B** Same, anterior end, frontal view **C** Same, ventro-caudal shield, frontal view **D**–**F** Paratypes (CAS 129027p), ventro-caudal shields. Bars **A** 1.2 mm **B, E** 0.6 mm **C** 0.5 mm **D** 0.8 mm **F** 0.9 mm.

Holotype (CAS 129027h) with integument almost completely removed, some fragments remain in mid-body; body wall broken midventrally and dorsally (Fig. 3A). Integment and ventro-caudal shield pale brown, body wall whitish, introvert darker than abdomen. Body papillae mostly removed along with integument; papillae probably homogeneously distributed throughout body. Body 13 mm long, 5 mm wide, about 28 segments; left ventro-caudal shield 2.0 mm long, 2.2 mm wide.

Prostomium minute globose, ovoid, paler than peristomium. Peristomium small, oval, papillae lost. Mouth oval, small slightly wider than prostomium, barely projected, covered by papillae (Fig. 3B).

First three chaetigers with about 16 falcate, tapered introvert hooks per bundle, each with subdistal dark areas, slightly paler distally. Genital papillae not seen. Pre-shield region with 7 segments; capillary chaetae not seen (some paratypes with capillaries in 2–4 segments).

Ventro-caudal shield completely covered by a thin, delicate coating of adhered fine sediment particles, mostly removed from shield; suture not visible (Fig. 3C). Anterior margins rounded; anterior depression deep; anterior keels not exposed. Ribs barely defined, concentric lines not visible. Lateral margins rounded, expanded medially, reduced posteriorly. Fan with a deep wide median notch, projected beyond the margins of posterior corners.

Marginal chaetal fascicles include 10 lateral and only 4 short, small posterior ones (others probably broken; some paratypes with up to six posterior bundles); lateral bundles with 7–8 chaetae each, posterior bundles with 5–6. Peg chaetae not visible (one paratype with minute, barely visible peg chaetae). Additional chaetal fascicles not visible.

Branchiae lost. Interbranchial filaments lost. Branchial plates convergent, anteriorly expanded, rounded. Some paratypes with broken body wall include oocytes, each about 200 µm.

##### Variation.

All paratypes have shields without sediment particles firmly adhered or concentric lines (Fig. 3D–F). The anterior depression is deep, the anterior margins are projected, and the radial ribs are progressively better developed as growth proceeds. The posterior, median notch is well developed in all stages.

##### Etymology.

The specific name is derived from the Latin adjective *nudus (a, um)*: naked, to indicate that unlike other species in the genus, its shield does not have firmly adhered sediment particles on it. The epithet is in the genitive case.

##### Remarks.

*Caulleryaspis nuda* sp. n. is unique because its ventro-caudal shield is soft as typical for the genus, but instead of having a thick sediment particles cover, it has a very thin layer made by loosely adhered, fine sediment particles, which can be easily eroded or brushed off. However, the general shield outline of *Caulleryaspis nuda* resembles the one present in *Sternaspis williamsae* sp. n. (see below), and the latter can even incorporate some sediment particles, but they differ because in *Caulleryaspis nuda*, the shield does not have a thin, stiff, yellowish layer but rather a convex, delicate, pliable margin.

##### Distribution.

Only known from off Oregon, U.S.A., in 2519 m depth.

#### Key to species of *Caulleryaspis* Sendall & Salazar-Vallejo, 2013

**Table:** 

1	Shield with sediment particles firmly adhered; shield surface not visible	2
–	Shield without firmly adhered sediment particles; shield surface visible	*Caulleryaspis nuda* sp. n. (Northeastern Pacific, off Oregon)
2(1)	Shield with anterior depression deep; peg chaetae robust	3
–	Shield with anterior depression shallow; peg chaetae indistinct	*Caulleryaspis laevis* (Caullery, 1944) (Indonesia)
3(2)	Shield with anterior margins angular; peg chaetae forming thick, large spines	*Caulleryaspis gudmundssoni* Sendall & Salazar-Vallejo, 2013 (North Atlantic, Iceland)
–	Shield with anterior margins rounded; peg chaetae forming thin, small spines	*Caulleryaspis fauchaldi* sp. n. (Northeastern Pacific, Oregon to California)

#### Sternaspis

Otto, 1821

http://species-id.net/wiki/Sternaspis

##### Type species.

*Sternaspis thalassemoides* Otto, 1821, by monotypy.

##### Diagnosis.

Sternaspids with introvert hooks falcate, tapered. Pre-shield region with 7 segments. Ventro-caudal shield stiff, usually with abundant sediment particles loosely adhered; with well-developed radial ribs and concentric lines. Branchial filaments arranged in discrete branchial plates.

#### Sternaspis annenkovae sp. n.

http://zoobank.org/4A4E8760-CD4A-472F-9765-D8B1F07AD0DB

http://species-id.net/wiki/Sternaspis_annenkovae

Figure 4

Sternaspis scutata : Levenstein 1961: 167, 1966: 59 (*non*Ranzani 1817, *partim*)

##### Type material.

**Northwestern Pacific Ocean**. Holotype (ZIRAS 50602) and 11 paratypes (ZIRAS 50603), variably damaged, most with broken body wall and some broken by half, R.V. Vityaz, Sta. 2209 (49°46'01"N, 157°48'06"E), 3980–4070 m, grab Ocean, 1953 (five paratypes with introvert variably exposed and damaged, 6.0–11.0 mm long, 4.0–6.0 mm wide, ventral shield left plate 2.0–2.8 mm long, 2.1–3.0 mm wide; oocytes yellowish, still in ovary, about 200 µm; paratypes without introvert but abdomen well preserved, 6–12 mm long, 4.0–8.0 mm wide, ventral shield left plate 2.0–2.8 mm long, 2.3–3.5 mm wide; oocytes in ovary about 200 µm).

##### Description.

**Figure 4.**
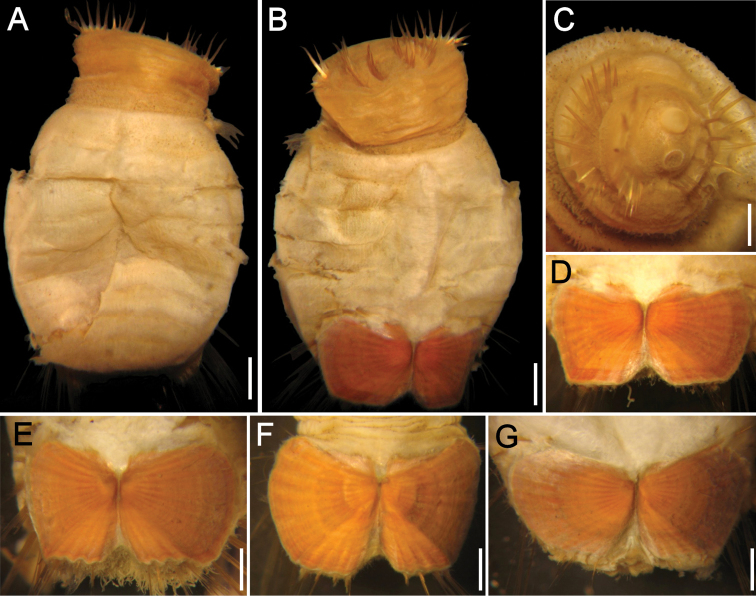
*Sternaspis annenkovae* sp. n. **A** Holotype (ZIRAS 50602), dorsal view **B** Same, ventral view **C** Same, ventro-caudal shield **D** Paratype, anterior end, oblique frontal view **E**–**G** Ventro-caudal shields of three paratypes. Bars: **A, B** 1.3 mm, **C, G** 1.1 mm, **D** 0.7 mm, **E** 0.8 mm, **F** 1 mm.

Holotype (ZIRAS 50602) with body bi-colored; introvert not fully exposed, pale brown, abdomen whitish (Fig. 4A), ventro-caudal shield pale brick red (Fig. 4B, D). Introvert finely papillose, abdomen papillae mostly eroded, some retained in folds or around branchial region. Body 10 mm long, 7 mm wide, abdomen 8 mm long, left ventro-caudal shield plate 2.0 mm long, 2.7 mm wide.

Prostomium hemispherical (Fig. 4C), (similar in paratypes; slightly acute in one paratype), projected, slightly larger than mouth, with same pigmentation as introvert. Eyespots not seen. Peristomium rounded with abundant papillae, extended laterally over prostomium and ventrally to margin of first chaetiger.

Introvert chaetigers with 10–11 golden barely falcate hooks, each with subdistal dark areas but tips mostly eroded (Fig. 4C). Genital papillae small, digitate, with same pigmentation than introvert, barely visible in intersegmental groove between segments 7 and 8.

Anterior abdomen with 7 segments, papillae mostly eroded, some remaining in body depressions or around branchial region, but not arranged in series or groups. Capillaries not seen (two paratypes with capillaries in first two segments, two per bundle).

Ventro-caudal shield with lateral plates divergent, surface with ribs and concentric lines, the latter less pronounced, barely banded; suture visible throughout shield. Anterior margins rounded, midventral depression shallow (Fig. 4C). Lateral margins gently rounded, not expanded posteriorly. Fan markedly notched, barely projected beyond poorly developed posterior corners, margin barely crenulated.

Marginal chaetal fascicles mostly broken off (Fig. 4B, C), 10 lateral ones with chaetae along an oblique series, and 7 posterior fascicles with chaetae in linear arrangement. Peg chaetae not visible.

Branchiae mostly lost (few remaining in paratypes, spirally bent); interbranchial papillae long, straight, often with fine sediment particles. Branchial plates observed in some paratypes, narrow, anteriorly rounded, wider than rest of branchial plate, with 8–9 filaments per series.

##### Etymology.

This species name is after the late Dr. Nadezhda P. Annenkova, in recognition of her many publications on polychaetes, and for her efforts to build a strong taxonomic tradition in the early to mid XX century in Russia. The epithet is a noun in the genitive case.

##### Variation.

The introvert is always pale brown, darker than the abdomen. Introvert chaetigers have 11–13 hooks per bundle. The ventro-caudal shield is pale brick red or dirty orange, ribs and concentric lines are always visible but variably developed; anterior margins are rounded to barely acute (Fig. 4E–G); fan markedly notched, margin barely crenulated to markedly crenulated, barely projected beyond the posterior corners. The inner margins of each lateral shield plate are fused along 1/2 to 1/3 of its length, resulting in a divergent or markedly notched fan. The shield chaetal bundles are difficult to count in holotype; paratypes with 9 lateral, and 6–7 posterior bundles.

##### Remarks.

*Sternaspis annenkovae* sp. n. is unique among the species in the genus and two features separate it. First, the body is bi-colored having a darker introvert and a pale abdomen, whereas in all other species the introvert is usually of the same color than the abdomen, slightly paler or even transparent. It is true that sometimes the sediment-filled gut can be displaced towards the introvert, making it look darker than the posterior region, but this can be noticed by transparency of the introvert’s body wall, whereas in *Sternaspis annenkovae* the pigmentation is widespread and homogeneous in the introvert. Second, the lateral shield plates are divergent and separated throughout its posterior region such that the fan is markedly notched. Some *Sternapsis* species can have a more eroded shield in older specimens, whereas the younger specimens are less eroded; however, in *Sternaspis annenkovae* the markedly notched fan is evident even in small specimens, rendering it a consistent feature, not significantly modified during growth. The other three deep-water species (*Sternaspis maior*, *Sternaspis princeps*, and *Sternaspis rietschi*) do not have a median notch or, if present, as in *Sternaspis maior*, it is rather shallow. Further, there are two species that have shields with deeply notched fans, especially in larger specimens as shown elsewhere (Sendall and Salazar-Vallejo 2013): *Sternaspis costata* von Marenzeller, 1879 and *Sternaspis islandica* Malmgren, 1867. These species differ by having a wider fan such that the larger ribs form an angle of 100–120° whereas in *Sternaspis annenkovae* the fan is narrower and the larger ribs form an angle of 85–90°. However, as indicated in the key below *Sternaspis annenkovae* resembles *Sternaspis fossor* Stimpson, 1853 because their shields have radial ribs and concentric lines, fans with a deep, median notch, and poorly defined posterior corners. They differ because in *Sternaspis annenkovae* the introvert is darker than the rest of the body and its shield is wider anteriorly, whereas in *Sternaspis fossor* the introvert is paler or with similar pigmentation than the rest of the body, and the shield is wider medially.

On the other hand, *Sternaspis annenkovae* resembles *Sternaspis williamsae* sp. n. (described below) because in the ventro-caudal shields of both species the fan is narrower than the corresponding anterior margins. They differ because in *Sternaspis annenkovae* concentric lines are better developed, the fan is medially discontinuous, and the prostomium is larger than the mouth, whereas in *Sternaspis williamsae* the concentric lines are poorly developed, the fan is medially continuous, and the prostomium is smaller than the mouth.

##### Distribution.

Only known from the type locality in the Northwestern Pacific Ocean, east off northern Kurile Islands, in 3980–4070 m depth. Levenstein (1961, 1966) deep water records from the Bering Sea could belong to this species but her materials were not found.

#### Sternaspis maureri sp. n.

http://zoobank.org/22EEC1F6-2325-4E44-A66E-943CD62E0D88

http://species-id.net/wiki/Sternaspis_maureri

Figures 5 , 6

Sternaspis fossor : Maurer and Williams 1988: 694 (*non*Stimpson 1853).
Sternaspis scutata : Kirkegaard 1996: 71–72 (*non*Ranzani 1817).

##### Type material.

**Eastern Pacific, Peru**. Holotype (LACM 5679), and two paratypes (LACM 5680), R.V. Anton Bruun, Cruise 11, Sta. 100 (08°16S, 81°05W), Menzies trawl, 6156–6489 m, 16 Oct. 1965 (paratypes anterior or posterior fragments; ventro-caudal shield with fan slightly narrower than anterior margins; margin crenulated, median and lateral notches well developed). Six paratypes (LACM 5681), R.V. Anton Bruun, Cruise 11, Sta. 72 (08°25S, 81°05W), Menzies trawl and Beam trawl, 6220–6052 m, 12 Oct. 1965 (one complete, one broken into two pieces, two fragmented introverts. Complete 8.0 mm long, 2.3 mm wide, left shield plate 2.5 mm long, 2.2 mm wide. Shield with margin narrower than anterior margins, margin smooth, paler, median notch shallow, lateral notches well developed; other remaining shield plates 2.0–2.5 mm long, 2.2–2.5 mm wide). Three paratypes (LACM 5682), R.V. Anton Bruun, Cruise 11, Sta. 77 (08°22S, 81°02W), Menzies trawl and Beam trawl, 6052–6260 m, 13 Oct. 1965 (two broken in several pieces; one complete with most shield chaetal bundles broken. Complete with introvert partly evaginated, 7.0 mm long, 2.5 mm wide; left shield plate 2.4 mm long, 2.1 mm wide. Other specimens with left shield plate 2.0–2.5 mm long, 2.3–2.5 mm wide. Fan projected, smooth in smaller specimens, crenulated in larger ones. Median notch visible, smaller than lateral notches; better developed posterolateral corners).

##### Additional material.

**Eastern Pacific.** One specimen (LACM 5684), R.V. Anton Bruun, Cruise 11, Sta. 36 (05°43'S, 82°01'W), Menzies trawl, 5047 m, 5 Oct. 1965 (broken into two portions, body wall broken, shield broken, one plate lost, the other missing a lateral part). Eight specimens (LACM 5686), R.V. Anton Bruun, Cruise 11, Sta. 69 (06°19'S, 81°49'W), Beam trawl, 5750 m, 11 Oct. 1965. (label with question mark about stations 55 or 69; herein regarded as part of station 69; two anterior fragments, one posterior fragment, two detached shields, three detached shield plates). 10 specimens (LACM 5683), R.V. Anton Bruun, Cruise 11, Sta. 94 (08°21'S, 81°25'W), Menzies trawl, 1296–1317 m, 14 Oct. 1965 (one broken into two pieces; six with introvert invaginated, three with introvert evaginated; complete specimens 1.8–4.8 mm long, 1.3–2.9 mm wide; left shield plate 0.4–1.0 mm long, 0.5–1.0 mm wide. Other specimens 2.0–4.2 mm long, 1.5–2.8 mm wide; left shield plate 0.5–1.0 mm long, 0.5–0.9 mm wide. Ventro-caudal shield fan starts being smooth or with a poorly defined posterior margin into a crenulated margin in larger specimens. All have the fan narrower than the anterior margin; median notch not developed, lateral notches well developed). One specimen (LACM 5685), R.V. Anton Bruun, Cruise 11, Sta. 98 (08°24'S, 81°15'W), Menzies trawl, 6052–5989 m, 15 Oct. 1965 (body wall broken, gut broken in pieces. Ventro-caudal shield with fan narrower than anterior margins, fan margin smooth, projected beyond the poorly defined posterolateral corners. Left shield plate 2.5 mm long, 2.3 mm wide). Two specimens (LACM 5689), R.V. Anton Bruun, Cruise 11, Sta. 98? (08°24'S, 81°15'W), Campbell grab, 6052 m, 15 Oct. 1965 (label with “Sta. 86 or 98?” but Maurer & Williams did not include station 86; both specimens broken, without introvert, shields detached from the body or still fixed over abdomen. Detached shield left plate 3.2 mm long, 2.6 mm wide. Shield with fan narrower than anterior margins, projected, margin crenulated, lateral notches well defined). One specimen (LACM 5690), R.V. Anton Bruun, Cruise 11, Sta. 101 (08°13'S, 81°09'W), Menzies trawl, 1927–1997 m, 16 Oct. 1965 (ventro-caudal shield with fan narrower than anterior margins, fan margin smooth, projected beyond the poorly defined posterolateral corners; left shield plate 1.2 mm long, 1.2 mm wide). Two specimens (LACM 5688), R.V. Anton Bruun, Cruise 11, Sta. 113 (08°44'S, 80°45'W), Menzies trawl and Beam trawl, 5986–6134 m, 19 Oct. 1965 (larger specimen without introvert; the other very small apparently with introvert invaginated. Larger specimen with ventro-caudal shield with fan narrower than anterior margins, fan margin smooth, projected beyond the poorly defined posterolateral corners; left shield plate 2.2 mm long, 2.2 mm wide. Smaller specimen with shield with fan projected beyond the poorly defined posterolateral corners; margin covered by sediment particles). One specimen (LACM 5691), R.V. Anton Bruun, Cruise 11, Sta. 196 (09°01'S, 80°40'W), Menzies trawl, 4516–4383 m, 7 Nov. 1965 (body broken into two pieces, most of body in one, the shield in another. Ventro-caudal shield with fan narrower than anterior margins, fan margin barely crenulated, projected beyond the poorly defined posterolateral corners. Left shield plate 1.3 mm long, 1.2 mm wide). **Southwestern Pacific**. One specimen (ZMUC 0000), juvenile, R.V. Galathea, S off Adelaide, Sta. 556 (37°18'S, 138°43'E), 795 m, 6 Dec. 1951 (7 mm long, 3 mm wide, left shield plate 1.2 mm long, 1.0 mm wide). One specimen (ZMUC 0000), R.V. Galathea, S off New Zealand, Sta. 607 (44°18'S, 166°46'E), 3830 m, 17 Jan. 1952 (broken in two parts, partly dehydrated, and the shield is very fragmented, but most pieces remain on site; damage made by removing the sediment and papillae surrounding the anal peduncle lobe; right shield plate 1.8 mm long, 1.3 mm wide).

##### Description.

**Figure 5.**
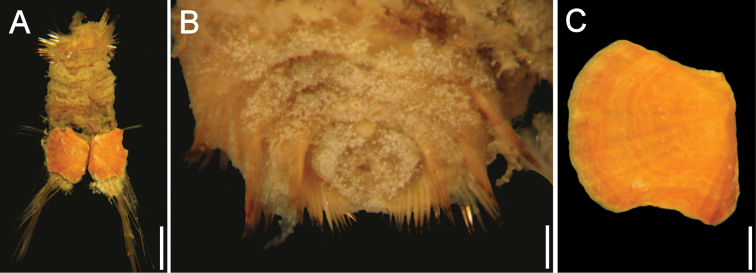
*Sternaspis maureri* sp. n., **A** Holotype (LACM 5679), ventral view, shield variably broken **B** Paratype (LACM 5680), anterior end, seen from above **C** Ventro-caudal shield, detached right part. Bars **A** 1.9 mm **B** 0.4 mm **C** 0.5 mm.

Holotype (LACM 0000) complete. Body wall broken and inner organs lost (Fig. 5A). Body brownish, integument papillose throughout body, dark brown (eroded leaving a paler body wall in smaller paratypes; larger ones with darker body wall). Body 7 mm long, 3 mm wide, about 29 segments; left shield plate 2.5 mm long, 2.1 mm wide.

Prostomium eroded, small, ovoid, paler than surrounding areas, smaller than mouth (Fig. 5B). Peristomium rounded, abundantly papillose (especially in some paratypes), papillae extended throughout introvert. Mouth rounded, papillose, slightly projected.

First three chaetigers with 12–14 bronze, slightly falcate hooks per bundle, each with subdistal dark areas (up to 16–18 in some paratypes). Genital papillae lost (pale, blunt, short lobes in some paratypes, from the intersegmental groove between segments 7 and 8).

Pre-shield region with 7 segments, with papillae mostly eroded from segmental ridges, but present in intersegmental furrows or along some areas, homogeneously distributed. Short, about 4–5 capillary chaetae in some segments.

Ventro-caudal shield dark orange, with ribs partly eroded, concentric lines poorly developed; suture distinct throughout shield (Fig. 5A, C). Anterior margins rounded (broken in Fig. 5A), anterior depression shallow; anterior keels not exposed, barely developed. Lateral margins rounded, reduced posteriorly. Fan truncate, one-half to two-thirds as wide as anterior margins width, slightly projected beyond posterior posterior shield corners, median notch shallow, barely developed, lateral notches deeper, better developed, fan margin smooth to barely crenulated (paler in some paratypes).

Marginal chaetae fascicles damaged; eight or nine lateral ones (10 in one paratype Sta. 72), chaetae ovally arranged, and six posterior ones (paratypes more damaged). Peg chaetae visible, not broken, but detached, and a single long, delicate chaetae.

Branchial plate missing (one paratype (Sta. 72) with one plate left but no branchial or interbranchial filaments left; branchial plate ovoid, tapering anteriorly; another paratype from the same station with branchial plates anteriorly converging).

##### Variation.

**Figure 6.**
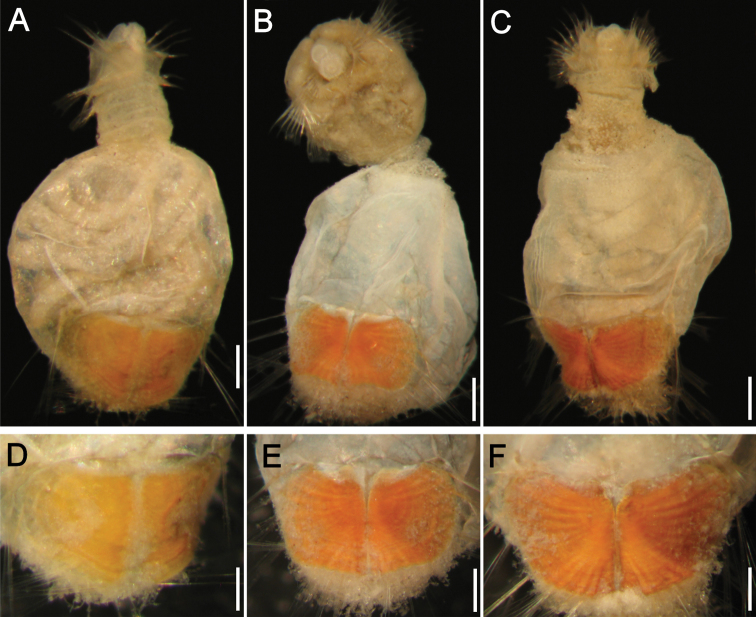
*Sternaspis maureri* sp. n., non-type specimens (Sta. 94 LACM 0000) **A**–**C** Ventral view of complete specimens **D**–**F** Ventro-caudal shields of the corresponding specimens. Bars **A** 0.2 mm **B** 2.2 mm **C** 0.6 mm **D** 0.15 mm **E** 0.3 mm **F** 0.4 mm.

There are several ontogenetic changes in the ventro-caudal shield, although the anterior depression remains shallow throughout their development (Fig. 6). Smaller specimens (Fig. 6A, D) have yellowish or pale orange shield, with ribs barely visible. Medium-sized specimens have shields slightly darker, with ribs better defined but the main, diagonal rib is still poorly developed (Fig. 6B, E). In larger specimens, the shield becomes thicker, stronger and its ribs, including the diagonal one are better defined (Fig. 6C, F) and the lateral notches, over its posterior margin or fan, become deeper.

##### Etymology.

The species name is after Dr Don Maurer in recognition of his many publications, mostly on benthic ecological studies and especially dealing with polychaetes. He also studied the material employed for this description. The epithet is a noun in the genitive case.

##### Type locality.

W off Trujillo, Perú, 6156–6489 m depth.

##### Remarks.

*Sternaspis maureri* sp. n. resembles *Sternaspis annenkovae* sp. n. and *Sternaspis williamsae* because they all have ventro-caudal shield fans narrower than the width of the anterior margin. However, the shield in *Sternaspis maureri* sp. n. is more similar to the one found in *Sternaspis williamsae* because both are medially fused. Their main differences are in the relative shield’s size, radial ribs’ development, and on the relative depth of the fan’s lateral and median notches. In *Sternaspis maureri* the shield is proportionally larger (body length: shield length 7.0:2.5 mm, one paratype Sta. 77, 6.5:2.5 mm), the ribs are better developed, the median notch is shallow or very slightly developed, and the lateral notches are better developed, whereas in *Sternaspis williamsae* the shield is smaller (7.5:1.6 mm), the ribs are poorly developed, its median notch is deep or well defined, and the lateral notches are poorly developed or indistinct.

##### Distribution.

Abyssal sediments off Peru, Eastern Pacific, in 1296–6489 m water depths, and in the Southwestern Pacific in 795–3830 m.

#### Sternaspis uschakovi sp. n.

http://zoobank.org/493904C2-D6D2-47FC-95D0-0A0A2221218D

http://species-id.net/wiki/Sternaspis_uschakovi

Figures 7 , 8

Sternaspis scutata : Uschakov 1950: 215, 1953: 154 (distr., *non*Ranzani 1817, *partim*).
Sternaspis fossor : Fauchald 1972: 238–239 (*partim*, *non*Stimpson 1853); Méndez 2007: 609, 613–616 (lists) (*partim*, *non*Stimpson 1853).

##### Type material.

**Northwestern Pacific Ocean**. Holotype (ZIRAS 50604) and 15 paratypes (ZIRAS 50605), Okhotsk Sea, R.V. Gagara, Sta. 251 (55°13'N, 146°52'E), 592 m, 12 Sep. 1932 (four complete paratypes exposing their introvert 5–8 mm long, 4.0–5.5 mm wide, abdomen 3.5–5.0 mm long, left ventro-caudal shield plate 1.0–1.6 mm long, 1.5–2.0 mm wide).

##### Additional material.

**Northwestern Pacific Ocean**. 12 specimens (ZIRAS 50606) Okhotsk Sea, R.V. Gagara, Sta. 215 (49°25'N, 152°00'E), 1366 m, 8 Aug. 1932 (10 specimens with exposed introvert 10.0–13.0 mm long,4.0 mm wide (waist), abdomen 4.0–5.5 mm long, left ventro-caudal shield plate 2.0–2.5 mm long, 2.0–2.8 mm wide). Three specimens (ZIRAS 50607) juveniles, Okhotsk Sea, R.V. Gagara, Sta. 214 (49°23'N, 148°46'E), 1076 m, 6 Aug. 1932 (juveniles with a very thin shield; abdomen 4.5 mm long, one with introvert partially exposed 7 mm long). **Northeastern Pacific Ocean**. Oregon. Three specimens (CAS 149923) previously dried out, BMT-554 (47°49.9'N, 127°2.9'W), 2510 m, 8 Sep. 1971, A. Carey, coll. (8.0–10.5 mm long, 3–5 mm wide; left shield plate 2.2–2.5 mm long, 2.9–3.0 mm wide). **California.** Nine specimens (SIORAS 4214), R.V. Vityaz, Cruise 29, Sta. 4214 (35°01.5'N, 121°42.5'W), 1585 m, 6 Dec. 1958 (best preserved specimen 12 mm long, 3.4 mm wide (waist), abdomen 8.6 mm long). **Gulf of California**. One specimen (UNAM 7879), introvert broken, off Ensenada del Pabellón, Sinaloa, RV El Puma, Cruise Talud IV, Sta. 19 (24°15'18"N, 108°24'06"W), 1245 m, 25 Aug. 2000 (5 mm long, abdomen 2.8 mm wide; ventro-caudal shield reddish, bent dorsally, wider than abdomen; fan projected medially, posterior margin dentate, last lateral chaetal bundles about 3 mm long). One specimen (UNAM 7880), introvert broken, RV El Puma, Cruise Talud IV, Sta. 26 (24°56'24"N, 109°05'36"W), 1200–1274 m, 26 Aug. 2000 (5.5 mm long, abdomen 2 mm wide; ventro-caudal shield broken, reddish, bent dorsally, wider than abdomen; fan projected medially, posterior margin dentate; last lateral chaetal bundles about 3 mm long). Three specimens (UNAM 7884), two complete, introvert exposed; off Lechugilla Island, Sinaloa, RV El Puma, Cruise Talud IV, Sta. 34 (25°43'50"N, 109°53'59"W), 1240–1270 m, 17 Mar. 2001 (6.3–11.0 mm long, abdomen 3.0–4.5 mm wide; ventro-caudal shield reddish, bent dorsally, wider than abdomen in one specimen; fan projected medially, posterior margin dentate; last lateral chaetal bundles about 3.5 mm long). **Western Mexico.** Eight specimens (LACM 5347), juveniles, most with introvert invaginated or damaged, some with body wall broken, S off Cabo Corrientes, Jalisco, R.V. Velero IV, Sta. 13755 (19°51'30"N, 105°50'00"W), 2548 m, mud, Campbell grab, 18 Jan. 1970 (4.0–5.0 mm long, 2.0 mm wide; left shield plate 0.8–1.3 mm long, 1.5–1.8 mm wide).

##### Description.

**Figure 7.**
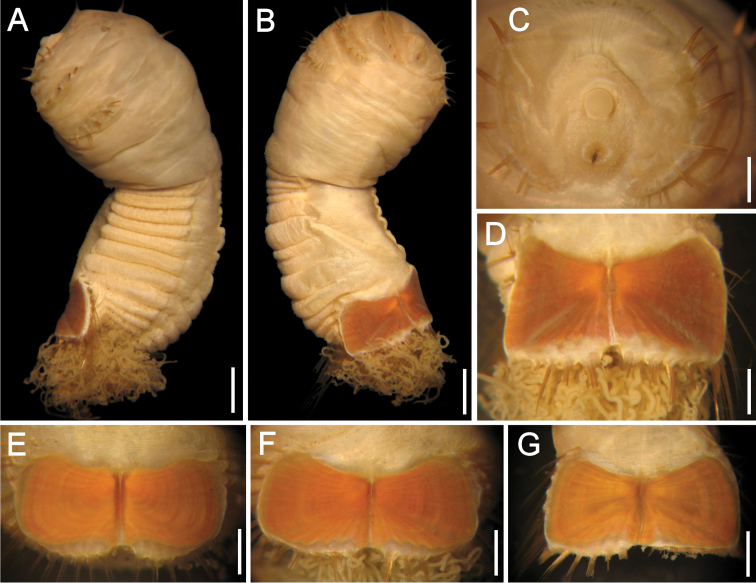
*Sternaspis uschakovi* sp. n. **A** Holotype (ZIRAS 50604), lateral view **B** Oblique ventral view **C** Anterior end, frontal view **D** Ventro-caudal shield, frontal view **E**–**F** Paratypes, ventro-caudal shields, frontal view **G** Non-type specimen (ZIRAS 50606), ventro-caudal shield, frontal view. Bars: **A** 1.7 mm **B** 1.2 **C** 0.8 mm **D** 1.5 mm **E** 0.5 mm **F** 0.9 mm **G** 1.2 mm.

Holotype (ZIRAS 50604) with body anteriorly swollen, slightly darker than posterior region; introvert fully exposed, pale, abdomen creamy (Fig. 7A), ventro-caudal shield dirty reddish, with orange central areas (Fig. 7B, D). Body 14 mm long, 5 mm wide (mid body constriction), abdomen 9 mm long, left ventro-caudal shield plate 2.2 mm long, 2.7 mm wide. Introvert mostly smooth, barely papillose, abdomen papillae mostly eroded, some retained in folds or around branchial region.

Prostomium hemispherical (Fig. 7C); projected, with same pigmentation as introvert. Eyespots not seen. Peristomium round with abundant papillae restricted to peripheral areas around the mouth, barely reaching margin of first chaetiger.

Introvert chaetigers with 9–11 golden, barely falcate hooks, each with subdistal dark areas but tips mostly eroded (Fig. 7C). Genital papillae small, thin, blunt, with same pigmentation than introvert, in intersegmental groove between segments 7 and 8.

Anterior abdomen with 7 segments, lateral lobes well-defined by contraction, dorsal area bare, converging posteriorly, ventral area bare, more or less parallel; papillae mostly eroded, some remaining in branchial region, but not arranged in series or groups. Capillaries not seen.

Ventro-caudal shield with lateral plates slightly bent dorsally, making them look quadrate in frontal view (Fig. 7D) but each plate wider than long (Fig. 7B); suture visible throughout shield. Ribs barely developed, concentric lines poorly developed but present. Anterior margins barely rounded, midventral depression shallow. Lateral margins gently rounded, expanded posteriorly. Fan projected medially beyond posterior corners, margin denticulate.

Marginal chaetal fascicles mostly broken off (Fig. 7A, B, D), 9 lateral ones with chaetae along an oblique series, and 6 posterior fascicles with chaetae in linear arrangement. Peg chaetae not visible (some paratypes have them).

Branchiae still attached, abundant, spirally bent filaments; interbranchial papillae long, spirally bent, with fine sediment particles. Branchial plates observed in some paratypes, wide, progressively wider towards anterior margin, with 7–8 filaments per series.

##### Etymology.

This species is being named after the late Dr. Pavel V. Uschakov as a modest homage to his monographic publications, especially those regarding the Okhotsk Sea fauna, and by his other numerous publications on polychaetes. The epithet is a noun in the genitive case.

##### Variation.

**Figure 8.**
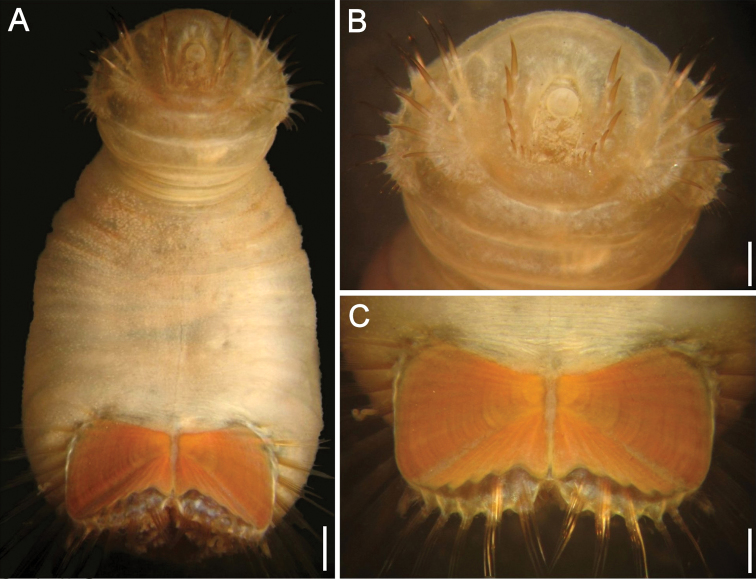
*Sternaspis uschakovi* sp. n., specimen from off Central California, U.S.A. (SIORAS 4214) **A** Frontal view, body ventrally bent **B** Anterior end, frontal view **C** Ventro-caudal shield, frontal view. Bars: **A** 1 mm **B** 0.5 mm **C** 0.6 mm.

The introvert is markedly swollen, like in the holotype, in one paratype and in some non-type specimens. Introvert chaetigers with 9–10 hooks per bundle. The ventro-caudal shield is dirty orange to pale brick red, ribs and concentric lines are always visible but variably developed; anterior margins rounded to barely acute (Fig. 7E–G); fan markedly projected medially, with shallow lateral notches, margin denticulate rarely reaching the level of the posterior corners. The inner margins of each lateral shield plate are fused along most of its length. The shield chaetal bundles are difficult to count but there are 9 lateral and 7 posterior bundles in better preserved paratypes. On the other hand, the specimens from the Northeastern Pacific have bodies with larger, more abundant papillae (Fig. 8A), and they are also evident in the mouth region (Fig. 8B); the ventro-caudal shield (Fig. 8C), however, is very similar to the specimens from the Northwestern Pacific. We regard the apparent difference in integument papillation as a result of different sampling and sorting procedures and not as a significant, diagnostic difference. Therefore, we conclude they are conspecifics.

##### Remarks.

*Sternaspis uschakovi* sp. n. differs from other deep-sea *Sternaspis* species because its ventro-caudal shield is medially projected, but resembles two other species having a ventro-caudal shield with denticulated posterior margin: *Sternaspis africana* Augener, 1918, and *Sternaspis andamanensis* Sendall & Salazar-Vallejo, 2013. However, these two species have been found in shallow water (5–70 m), whereas *Sternaspis uschakovi* was found in water depths of 592–2548 m. There are two other differences when this new species is compared to the other two; first, the midventral depression is shallow in the new species, resembling *Sternaspis africana*, whereas it is deep in *Sternaspis andamensis*; and second, the fan posterior margin has two lateral deep notches in *Sternaspis andamanensis* whereas in *Sternaspis uschakovi* and in *Sternaspis africana* the fan is not so markedly notched laterally. Further, as indicated in the key below, *Sternaspis uschakovi* is more similar to *Sternaspis andamanensis* but they can be easily separated because in *Sternaspis uschakovi* there are about 10 dark introvert hooks per bundle, whereas in *Sternaspis andamanensis* there are about 15 pale introvert hooks per bundle.

##### Distribution.

Okhotsk Sea in 592–1366 m, California in 1585 m, Gulf of California in 1200–1274 m, and off Western Mexico in 2548 m, in mud or muddy sands.

#### Sternaspis williamsae sp. n.

http://zoobank.org/83507969-B8CA-4028-9521-C76F676486F0

http://species-id.net/wiki/Sternaspis_williamsae

Figures 9 , 10

Sternaspis fossor : Hartman 1963: 59; Fauchald and Hancock 1981: 35 (*partim*, *non*Stimpson 1853).

##### Type material.

**Northeastern Pacific, Oregon**. Holotype (LACM 5353), and five paratypes (LACM 5354), off Columbia River, R.V. Acona, Sta. AD 33, NAD 21 (44°30.0'N, 125°34.0'W – 44°39.0'N, 125 33.2 W), 2800 m, 25 Jan. 1963 (paratypes 2.2–9.8 mm long, 1.4–4.5 mm wide, abdomen 2.2–6.0 mm long; left shield plate 0.9–1.8 mm long, 0.8–2.2 mm wide).

##### Additional material.

**Northeastern Pacific, Oregon**. One specimen (CAS 129027w), Sta. BMT 557 (48°9.0'N, 127°4.2'W), 2519 m, 10 Sep. 1971, A. Carey, coll. (9 mm long, 4.8 mm wide; left shield plate 2.0 mm long, 1.8 mm wide). One specimen (LACM 5359), too damaged, broken into two pieces, shield almost completely lost, off Columbia River, R.V. Acona, Sta. AD 9, NAD 21 (44°36.4'N, 125°24.8'W), 2800 m, 13 Aug. 1962. One specimen (LACM 5355), off Columbia River, R.V. Acona, Sta. AD 32, NAD 19 (44°38.6'N, 125°20.1'W – 44°37.6'N, 125°21.0'W), 2400 m, 25 Jan. 1963 (4.5 mm long, 2 mm wide, abdomen 3 mm long; left shield plate 1.1 mm long, 0.9 mm wide). Two specimens (LACM 5358), juveniles, Cascadia Abyssal Plain, W off Yaquina Bay, R.V. Acona, Sta. AD 141, NAD11B NAD 19 (44°38.6'N, 125°20.1'W – 44°37.6'N, 125°21.0'W), 2400 m, clayey silt, 8 Apr. 1965 (3.5–4.0 mm long, 2.0–2.5 mm wide, abdomen 2.5–3.0 mm long; left shield plate 0.5–0.7 mm long, 0.5–0.9 mm wide). One specimen (LACM 5356), juvenile, too contracted, introvert damaged, Cascadia Abyssal Plain, W off Yaquina Bay, R.V. Acona, Sta. AD 148, NAD 12 (44°40.7'N, 125°10.0'W – 44°41.1'N, 125°10.0'W), 1000 m, 5 Jun. 1965 (genital papillae small, digitate; body 2 mm long, 2.2 mm wide, left shield plate 0.6 mm long, 0.8 mm wide). Five specimens (LACM 5357), juveniles, Cascadia Abyssal Plain, W off Yaquina Bay, R.V. Acona, Sta. AD 149, NAD 15 (44°41.2'N, 125°15.0'W – 44°91.9'N, 125°15.1'W), 1600 m, silty sand, 5 Jun. 1965 (0.7–4.5 mm long, 0.8–3.0 mm wide, abdomen 0.6–3.0 mm long; left shield plate 0.4–0.6 mm long, 0.5–0.9 mm wide). **Northeastern Pacific, Southern California, canyons.** Fifteen specimens (LACM 5363), juveniles, 4.9 km off Gull Island, Santa Cruz Island, R.V. Velero IV, Sta. 6808 (33°54'30"N, 119°47'22"W), green sandy mud, 878 m, 22 Dec. 1959 (3.0–5.0 mm long, 1.5–3.0 mm wide, abdomen 2.0–3.0 mm long; left shield plate 0.5–0.8 mm long, 0.5–0.8 mm wide). Five specimens (LACM 5364), two juveniles, 10.7 km off Ribbon Rock, Santa Catalina Island, R.V. Velero IV, Sta. 6828 (33°20'30"N, 118°39'05"W), green mud, 1246 m, 28 Jan. 1960 (3.0–6.5 mm long, 2.0–3.0 mm wide, abdomen 2.0–3.5 mm long; left shield plate 0.7–0.9 mm long, 0.8–1.1 mm wide). **BLM (= Bureau of Land Management) Baseline Study, Southern California Bight.** One specimen (LACM 5327), dried out, BLM 81202, R.V. Thomas G. Thompson, Sta. 812 (33°46.384’N, 119°35.818’W), 1419 m, olive green soupy mud, biological box core, rep. 2 BFI/ B3-5, 22 Aug. 1977, K. Fauchald, G. Jones, coll. One specimen (LACM 5328), bent over itself, body wall broken, BLM 81236, R.V. Thomas G. Thompson, Sta. 812 (33°46.25'N, 119°36.30'W), 1419 m, olive green soupy mud, biological box core, rep. 36 BFI/ B3-5, 31 Aug. 1977, K. Fauchald, G. Jones, coll. One specimen (LACM 5329), body wall broken, BLM 81237, R.V. Thomas G. Thompson, Sta. 812 (33°46.24'N, 119°36.25'W), 1419 m, olive green soupy mud, biological box core, rep. 37 BFI/ B3-5, 31 Aug. 1977, K. Fauchald, G. Jones, coll. (4 mm long, 3 mm wide, abdomen 3.5 mm long, left shield plate 1 mm long, 1 mm wide). Two specimens (LACM 5330), one with introvert invaginated, BLM 81238, R.V. Thomas G. Thompson, Sta. 812 (33°46.24'N, 119°36.26'W), 1419 m, olive green soupy mud, biological box core, rep. 38 BFI/ B3-5, 31 Aug. 1977, K. Fauchald, G. Jones, coll. (5 mm long, 2 mm wide, abdomen 3 mm long, left shield plate 1 mm long, 1 mm wide). One specimen (LACM 5331), BLM 81302, R.V. Thomas G. Thompson, Sta. 813 (33°46.24'N, 119°36.26'W), 1758 m, olive brown silty mud, biological box core, rep. 02 BFI/ B4-1, 23 Aug. 1977, K. Fauchald, G. Jones, coll. (abdomen 3 mm long, 3 mm wide; left shield plate 1 mm long, 1 mm wide). Three specimens (LACM 5332), one mature female, BLM 81303, R.V. Thomas G. Thompson, Sta. 813 (33°0.951'N, 119°1.987'W), 1756 m, olive brown silt, biological box core, rep. 03 BFI/ B4-1, 23 Aug. 1977, K. Fauchald, G. Jones, coll. (abdomen 1.8–2.5 mm long, 1.5–2.0 mm wide; left shield plate 0.4–0.8 mm long, 0.5–0.8 mm wide; oocytes 180–200 µm in diameter). Two specimens (LACM 5332a), dried-out, BLM 81305, R.V. Thomas G. Thompson, Sta. 813 (33°1.003'N, 119°1.977'W), 1758 m, olive brown silt, biological box core, rep. 05 BFI/ B4-1, 23 Aug. 1977, K. Fauchald, G. Jones, coll. Two specimens (LACM 5338), BLM 81402, R.V. Thomas G. Thompson, Sta. 814 (32°48.483'N, 119°7.909'W), 931 m, olive green silty mud with shells, biological box core, rep. 02 BFI/ B4-2, 24 Aug. 1977, K. Fauchald, G. Jones, coll. (5 mm long, 2.5–2.8 mm wide, abdomen 3.0–3.5 mm long, left shield plate 0.7–0.9 mm long, 0.7–0.9 mm wide). One specimen (LACM 5339), BLM 81404, R.V. Thomas G. Thompson, Sta. 814 (32°48.481'N, 119°7.872'W), 933 m, olive green silty mud, biological box core, rep. 04 BFI/ B4-2, 24 Aug. 1977, K. Fauchald, G. Jones, coll. (3 mm long, 2 mm wide, abdomen 2 mm long, left shield plate 0.6 mm long, 0.6 mm wide). Two specimens (LACM 5341), dried-out, BLM 81407, R.V. Thomas G. Thompson, Sta. 814 (32°48.465'N, 119°7.946'W), 922 m, olive green silty mud, biological box core, rep. 07 BFI/ B4-2, 24 Aug. 1977, K. Fauchald, G. Jones, coll. One specimen (LACM 5342), dried-out, BLM 81408, R.V. Thomas G. Thompson, Sta. 814 (32°48.440'N, 119°7.828'W), 940 m, olive green silty mud, biological box core, rep. 08 BFI/ B4-2, 24 Aug. 1977, K. Fauchald, G. Jones, coll. Two specimens (LACM 5343), dried-out, BLM 81418, R.V. Thomas G. Thompson, Sta. 814 (32°48.53'N, 119°7.89'W), 920 m, olive green silty mud, biological box core, rep. 18 BFI/ B4-2, 24 Aug. 1977, K. Fauchald, G. Jones, coll. Two specimens (LACM 5344), dried-out, BLM 81422, R.V. Thomas G. Thompson, Sta. 814 (32°48.48'N, 119°7.81'W), 920 m, olive green silty mud, biological box core, rep. 22 BFI/ B4-2, 24 Aug. 1977, K. Fauchald, G. Jones, coll. One specimen (LACM 5345), BLM 82502, 14 km S of Huntington Beach, R.V. Thomas G. Thompson, Sta. 825 (33°33.035'N, 118°0.820'W), 250 m, olive green gray silty clay mud, biological box core, rep. 2 BFI/ B6-2, 3 Feb. 1977, K. Fauchald, G. Jones, coll. (abdomen 3 mm long, 2.3 mm wide; left shield plate 0.8 mm long, 0.8 mm wide). One specimen (LACM 5346), dried-out, BLM 84005, R.V. Thomas G. Thompson, Sta. 840 (33°24.56'N, 119°30.12'W), 713 m, olive green silty mud, biological box core, rep. 5 BFI/ D2-1, 3 Feb. 1977, K. Fauchald, G. Jones, coll.

##### Description.

**Figure 9.**
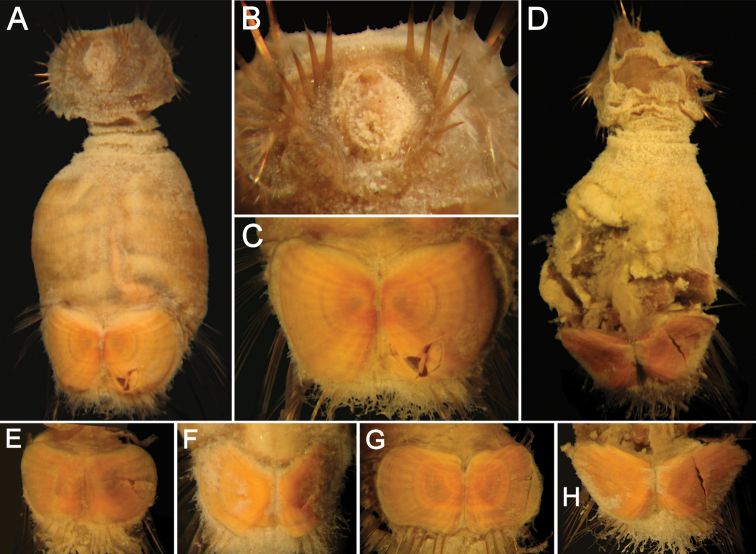
*Sternaspis williamsae* sp. n., holotype (LACM 5353) **A** Frontal view **B** Anterior end, oblique frontal view **C** Ventro-caudal shield **D** Largest paratype (LACM 5354), ventral view **E**–**H** Ventro-caudal shields, frontal view. Bars: **A, G** 0.9 mm **B, H** 1 mm **C** 0.5 mm **D** 1.2 mm **E** 0.4 mm **F** 0.6 mm.

Holotype (LACM 5353) with body brownish, paler over remaining integument with papillae (Fig. 9A). Introvert exposed, narrower than abdomen, covered with abundant papillae, eroded in some areas. Abdomen with integument smooth without papillae in swollen areas, otherwise with abundant small papillae. Body 7.5 mm long, 3.7 mm wide, abdomen 5.5 mm long; about 29 segments; left shield plate 1.6 mm long, 1.5 mm wide.

Prostomium small ovoid, paler distally, darker basally (Fig. 9B), smaller than mouth. Peristomium rounded, with a band of abundant papillae not reaching base of introvert hooks of chaetiger 1. Mouth oval, slightly projected, papillose.

First three chaetigers with 12–14 bronze, slightly falcate hooks per bundle, each with subdistal dark areas. Genital papillae lost, eroded from the intersegmental groove between segments 7 and 8 (not visible in paratypes either).

Pre-shield region with 7 segments, with papillae abundant in protected areas (some paratypes with papillae arranged in single transverse series per segment). Short, few capillary chaetae present in one segment (in up to three segments in paratypes).

Ventro-caudal shield pale orange (Fig. 9C), with ribs, concentric lines poorly developed; suture distinct throughout shield. Anterior margins rounded; anterior depression deep; anterior keels exposed (also exposed in two paratypes). Lateral margins rounded, reduced posteriorly. Fan truncate, half as wide as anterior margins width, slightly projected beyond posterior shield corners, median notch moderate, fan margin smooth (barely crenulated in two paratypes).

Marginal chaetal fascicles include 10 lateral ones; chaetae ovally arranged, and 5 posterior fascicles (6–7 in paratypes). Peg chaetae or associated capillaries not visible (neither in paratypes).

Branchiae lost in holotype (paratypes with abundant filaments, some 2–3 times thicker, and half as long as the others). Branchial plates parallel, anteriorly rounded.

##### Variation.

There are several modifications in the shield development with roughly defined ribs and the general outline has lateral margins rounded, medially expanded, some distortions were noticed probably due to sample handling since the body is rather delicate. The shield pigmentations is dark yellow in smaller paratypes (Fig. 9E) with radial ribs barely visible and no concentric lines; the posterior margin is smooth with the fan not projected beyond the posterior corners. Slightly larger paratypes have a better defined diagonal rib (Fig. 9F), but concentric lines appear later (Fig. 9G), together with slight lateral notches. The largest paratype was severely damaged (Fig. 9D), and its shield is almost completely detached and bent dorsally, such that its outline is apparently different (Fig. 9H); it has a well-developed diagonal rib but concentric lines are barely visible, and the lateral notches are not as distinct as in smaller specimens.

##### Early shield development.

**Figure 10.**
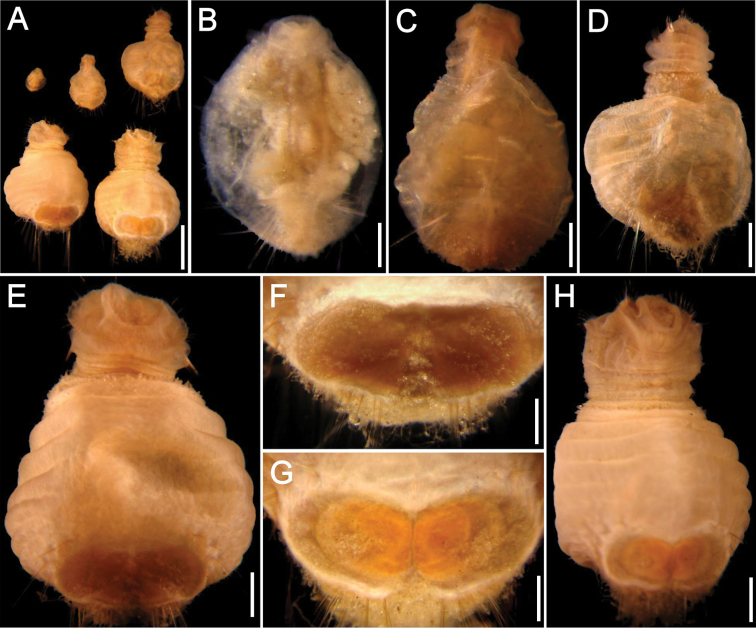
*Sternaspis williamsae* sp. n. Juveniles (LACM 5357) **A** Complete specimens, ventral view **B** Smallest juvenile, ventral view **C** Second specimen, ventral view **D** Intermediate-sized juvenile, ventral view **E** Second largest specimen, ventral view **F** Same, ventro-caudal shield, oblique illumination **G** Largest specimen, ventral view **H** Same, ventro-caudal shield, incident illumination. Bars: **A** 1.4 mm **B** 0.1 mm **C** 0.2 mm **D, G** 0.6 mm **E** 0.5 mm **F, H** 0.3 mm.

Very small juveniles show a transition in pigmentation and development of the ventro-caudal shield (Fig. 10A). The smallest specimen, being 0.7 mm long (Fig. 10B) has no pigmentation but chaetae are already arranged in lateral and posterior bundles, each with 1–3 chaetae. When the body doubles its size, being about 1.5 mm long (Fig. 10C) the shield has some sediment particles and becomes slightly more pigmented than in smaller specimens. The following stage, when the body reaches about 3 mm long (Fig. 10D), is not markedly different from the previous stage. Darker pigmentation and better defined shield margins are attained when the specimens are 3.5 mm long (Fig. 10E, oblique illumination resulted in a darker shield) but the ribs or the median notch are not well defined. These last two modifications apparently appear soon afterwards because in a similar-sized specimen (Fig. 10H), the shield (with a more incident angle) shows a dark yellow shield bordered by a paler region, and with better defined ribs and median notch (Fig. 10G).

##### Etymology.

This species is named after Susan Williams, in recognition of her taxonomic work on trichobranchids, and because she left some notes indicating that she regarded some of the materials herein included for this description as representing a different pattern which deserved a name. The epithet is a noun in the genitive case.

##### Type locality.

W off Yaquina Bay, Oregon, 2800 m depth.

##### Remarks.

*Sternaspis williamsae* sp. n. is very similar to *Sternaspis maior* Chamberlin, 1919 as redescribed elsewhere (Sendall and Salazar-Vallejo 2013) because both have shield with ribs and poorly-developed concentric lines. They differ, however, because of the relative development of some shield features. In *Sternaspis williamsae* the main ribs are moderately divergent, and the fan is narrower than that of the anterior margin, being up to half as wide as the anterior margin, whereas in *Sternaspis maior* the main ribs are markedly divergent, and the fan is as wide or wider than that of the anterior margin. On the other hand, *Sternaspis williamsae* is similar to *Sternaspis annenkovae* because both species have shields with fan narrower than that of the anterior margin. These two species differ because in the shield of*Sternaspis williamsae* the concentric lines are poorly developed, the fan is medially continuous, and its prostomium is smaller than the mouth, whereas in *Sternaspis annenkovae* concentric lines are better developed, the fan is medially discontinuous, and the prostomium is larger than the mouth. As stated above, *Sternaspis williamsae* resembles *Sternaspis maureri* because of their shields. They differ, however, in three main features. In *Sternaspis williamsae* the ribs are poorly developed, its median notch is deep or well defined, and its lateral notches are poorly developed or indistinct, whereas in *Sternaspis maureri* the ribs are better developed, its median notch is shallow or very slightly developed, and its lateral notches are better developed.

##### Distribution.

Only known from Oregon to California, 1000–2800 m depth.

#### Key to species of *Sternaspis* Otto, 1821

(Modified after Sendall and Salazar-Vallejo 2013)

**Table:** 

1	Ventro-caudal shield’s fan with a distinct median notch	2
–	Ventro-caudal shield’s fan continuous, without a distinct median notch	9
2(1)	Shield with radial ribs and concentric lines distinct	3
–	Shield with radial ribs distinct, concentric lines barely visible	7
3(2)	Fan with median notch shallow	4
–	Fan with median notch deep; shields usually with concentric bands	5
4(3)	Shield with distinct concentric bands; main rib and posterior corners directed posteriorly	*Sternaspis affinis* Stimpson, 1864 (NE Pacific Ocean)
–	Shield without concentric bands; posterior corners directed laterally	*Sternaspis scutata* (Ranzani, 1817) (Mediterranean Sea and NE Atlantic Ocean)
5(3)	Shield with posterior corners distinct	*Sternaspis costata* von Marenzeller, 1879 (NW Pacific Ocean)
–	Shield with posterior corners poorly-defined	6
6(5)	Introvert and body with similar pigmentation or introvert paler; shield widest medially	*Sternaspis fossor* Stimpson, 1853 (NW Atlantic Ocean)
–	Introvert and body with different pigmentation, introvert darker; shield widest anteriorly	*Sternaspis annenkovae* sp. n. (NW Pacific Ocean)
7(2)	Main ribs markedly divergent; fan as wide as anterior margins or wider	*Sternaspis maior* Chamberlin, 1919 (Eastern Pacific, Gulf of California)
–	Main ribs moderately divergent; fan half as wide as anterior margins	8
8(7)	Posterior margin truncate, with lateral notches; ribs well-developed	*Sternaspis maureri* sp. n. (Central Eastern Pacific)
–	Posterior margin with a median notch, lateral notches shallow or indistinct; ribs poorly developed	*Sternaspis williamsae* sp. n. (Northeastern Pacific)
9(1)	Fan margin crenulated, not projected posteriorly	10
–	Fan margin denticulate, medially projected	14
10(9)	Shield with ribs and concentric lines	11
–	Shield with ribs; concentric lines indistinct	*Sternaspis princeps* Selenka, 1885 (SW Pacific Ocean, New Zealand)
11(10)	Shield anterior margins rounded	12
–	Shield anterior margins acute	*Sternaspis spinosa* Sluiter, 1882 (Indonesia, Java)
12(11)	Shield with posterior corners distinct	13
–	Shield with posterior corners indistinct	*Sternaspis rietschi* Caullery, 1944 (Indonesia)
13(12)	Posterior corners barely projected beyond fan margin; introvert hooks thick, bronze	*Sternaspis thalassemoides* Otto, 1821 (NE Atlantic Ocean and Mediterranean Sea)
–	Posterior corners projected beyond fan margin; introvert hooks thin, golden	*Sternaspis thorsoni* Sendall & Salazar-Vallejo, 2013 (Indian Ocean, Arabian Gulf)
14(9)	Fan without lateral notches; body papillae arranged in distinct transverse rows	*Sternaspis africana* Augener, 1918 (Eastern Atlantic Ocean, Ghana to Angola).
–	Fan with lateral notches; body papillae distributed homogeneously, not arranged in transverse rows	15
15(14)	Introvert with about 15 pale hooks per bundle	*Sternaspis andamanensis* Sendall & Salazar-Vallejo, 2013 (Indian Ocean, Andaman Sea)
–	Introvert with about 10 dark hooks per bundle	*Sternaspis uschakovi* sp. n. (Northern Pacific Ocean)

## Supplementary Material

XML Treatment for Caulleryaspis

XML Treatment for Caulleryaspis fauchaldi

XML Treatment for Caulleryaspis nuda

XML Treatment for Sternaspis

XML Treatment for Sternaspis annenkovae

XML Treatment for Sternaspis maureri

XML Treatment for Sternaspis uschakovi

XML Treatment for Sternaspis williamsae
